# An Integral Representation of the Logarithmic Function with Applications in Information Theory

**DOI:** 10.3390/e22010051

**Published:** 2019-12-30

**Authors:** Neri Merhav, Igal Sason

**Affiliations:** The Andrew and Erna Viterbi Faculty of Electrical Engineering, Israel Institute of Technology Technion City, Haifa 3200003, Israel; merhav@ee.technion.ac.il

**Keywords:** integral representation, logarithmic expectation, universal data compression, entropy, differential entropy, ergodic capacity, SIMO channel, multivariate Cauchy distribution

## Abstract

We explore a well-known integral representation of the logarithmic function, and demonstrate its usefulness in obtaining compact, easily computable exact formulas for quantities that involve expectations and higher moments of the logarithm of a positive random variable (or the logarithm of a sum of i.i.d. positive random variables). The integral representation of the logarithm is proved useful in a variety of information-theoretic applications, including universal lossless data compression, entropy and differential entropy evaluations, and the calculation of the ergodic capacity of the single-input, multiple-output (SIMO) Gaussian channel with random parameters (known to both transmitter and receiver). This integral representation and its variants are anticipated to serve as a useful tool in additional applications, as a rigorous alternative to the popular (but non-rigorous) replica method (at least in some situations).

## 1. Introduction

In analytic derivations pertaining to many problem areas in information theory, one frequently encounters the need to calculate expectations and higher moments of expressions that involve the logarithm of a positive-valued random variable, or more generally, the logarithm of the sum of several i.i.d. positive random variables. The common practice, in such situations, is either to resort to upper and lower bounds on the desired expression (e.g., using Jensen’s inequality or any other well-known inequalities), or to apply the Taylor series expansion of the logarithmic function. A more modern approach is to use the replica method (see, e.g., in [1] (Chapter 8)), which is a popular (but non-rigorous) tool that has been borrowed from the field of statistical physics with considerable success.

The purpose of this work is to point out to an alternative approach and to demonstrate its usefulness in some frequently encountered situations. In particular, we consider the following integral representation of the logarithmic function (to be proved in the sequel),

(1) $$\begin{array}{c} \ln x=\int_{0}^{\infty }\frac{e^{-u}-e^{-ux}}{u}\;du,\;x>0. \end{array}$$

The immediate use of this representation is in situations where the argument of the logarithmic function is a positive-valued random variable, *X*, and we wish to calculate the expectation, E{lnX}. By commuting the expectation operator with the integration over *u* (assuming that this commutation is valid), the calculation of E{lnX} is replaced by the (often easier) calculation of the moment-generating function (MGF) of *X*, as

(2) $$\begin{array}{c} E\left\{\ln X\right\}=\int_{0}^{\infty }\left[e^{-u}-E\left\{e^{-uX}\right\}\right]\;\frac{du}{u}. \end{array}$$

Moreover, if $X_{1},\ldots ,X_{n}$ are positive i.i.d. random variables, then

(3) $$\begin{array}{c} E\left\{\ln\left(X_{1}+\ldots +X_{n}\right)\right\}=\int_{0}^{\infty }\left(e^{-u}-{\left[E\{e^{-uX_{1}}\}\right]}^{n}\right)\;\frac{du}{u}. \end{array}$$

This simple idea is not quite new. It has been used in the physics literature, see, e.g., [1] (Exercise 7.6, p. 140), [2] (Equation (2.4) and onward) and [3] (Equation (12) and onward). With the exception of [4], we are not aware of any work in the information theory literature where it has been used. The purpose of this paper is to demonstrate additional information-theoretic applications, as the need to evaluate logarithmic expectations is not rare at all in many problem areas of information theory. Moreover, the integral representation (1) is useful also for evaluating higher moments of lnX, most notably, the second moment or variance, in order to assess the statistical fluctuations around the mean.

We demonstrate the usefulness of this approach in several application areas, including entropy and differential entropy evaluations, performance analysis of universal lossless source codes, and calculations of the ergodic capacity of the Rayleigh single-input multiple-output (SIMO) channel. In some of these examples, we also demonstrate the calculation of variances associated with the relevant random variables of interest. As a side remark, in the same spirit of introducing integral representations and applying them, Simon and Divsalar [5,6] brought to the attention of communication theorists useful, definite-integral forms of the *Q*–function (Craig’s formula [7]) and Marcum *Q*–function, and demonstrated their utility in applications.

It should be pointed out that most of our results remain in the form of a single- or double-definite integral of certain functions that depend on the parameters of the problem in question. Strictly speaking, such a definite integral may not be considered a closed-form expression, but nevertheless, we can say the following.

(a) In most of our examples, the expression we obtain is more compact, more elegant, and often more insightful than the original quantity.
(b) The resulting definite integral can actually be considered a closed-form expression “for every practical purpose” since definite integrals in one or two dimensions can be calculated instantly using built-in numerical integration operations in MATLAB, Maple, Mathematica, or other mathematical software tools. This is largely similar to the case of expressions that include standard functions (e.g., trigonometric, logarithmic, exponential functions, etc.), which are commonly considered to be closed-form expressions.
(c) The integrals can also be evaluated by power series expansions of the integrand, followed by term-by-term integration.
(d) Owing to Item (c), the asymptotic behavior in the parameters of the model can be evaluated.
(e) At least in two of our examples, we show how to pass from an *n*–dimensional integral (with an arbitrarily large *n*) to one– or two–dimensional integrals. This passage is in the spirit of the transition from a multiletter expression to a single–letter expression.

To give some preliminary flavor of our message in this work, we conclude this introduction by mentioning a possible use of the integral representation in the context of calculating the entropy of a Poissonian random variable. For a Poissonian random variable, *N*, with parameter λ, the entropy (in nats) is given by

(4) $$\begin{array}{c} H\left(\lambda \right)=-E\left\{\ln\left(\frac{e^{-\lambda }\lambda^{N}}{N!}\right)\right\}=\lambda -\lambda \ln\lambda +E\left\{\ln N!\right\}, \end{array}$$

where the nontrivial part of the calculation is associated with the last term, E{lnN!}. In [8], this term was handled by using a nontrivial formula due to Malmstén (see [9] (pp. 20–21)), which represents the logarithm of Euler’s Gamma function in an integral form (see also [10]). In Section 2, we derive the relevant quantity using (1), in a simpler and more transparent form which is similar to [11] ((2.3)–(2.4)).

The outline of the remaining part of this paper is as follows. In Section 2, we provide some basic mathematical background concerning the integral representation (2) and some of its variants. In Section 3, we present the application examples. Finally, in Section 4, we summarize and provide some outlook.

## 2. Mathematical Background

In this section, we present the main mathematical background associated with the integral representation (1), and provide several variants of this relation, most of which are later used in this paper. For reasons that will become apparent shortly, we extend the scope to the complex plane.

**Proposition** **1.** (5) lnz=∫0∞e−u−e−uzudu,Re(z)≥0.

**Proof.**  (6) $$\begin{array}{cc} \ln z & =\left(z-1\right)\int_{0}^{1}\frac{dv}{1+v(z-1)} \end{array}$$

(7) $$\begin{array}{cc} \; & =\left(z-1\right)\;\int_{0}^{1}\int_{0}^{\infty }e^{-u[1+v(z-1)]}\;du\;dv \end{array}$$

(8) $$\begin{array}{cc} \; & =\left(z-1\right)\;\int_{0}^{\infty }e^{-u}\;\int_{0}^{1}e^{-uv(z-1)}\;dv\;du \end{array}$$

(9) $$\begin{array}{cc} \; & =\int_{0}^{\infty }\frac{e^{-u}}{u}\;\left[1-e^{-u(z-1)}\right]\;du \end{array}$$

(10) $$\begin{array}{cc} \; & =\int_{0}^{\infty }\frac{e^{-u}-e^{-uz}}{u}\;du, \end{array}$$

where Equation (7) holds as Re{1+v(z−1)}>0 for all v∈(0,1), based on the assumption that Re(z)≥0; (8) holds by switching the order of integration. □

**Remark** **1.** *In [12] (p. 363, Identity (3.434.2)), it is stated that*

(11) ∫0∞e−μx−e−νxxdx=lnνμ,Re(μ)>0,Re(ν)>0.

*Proposition 1 also applies to any purely imaginary number, z, which is of interest too (see Corollary 1 in the sequel, and the identity with the characteristic function in (14)).*

Proposition 1 paves the way to obtaining some additional related integral representations of the logarithmic function for the reals.

**Corollary** **1.** *([12] (p. 451, Identity 3.784.1)) For every x>0,*

(12) $$\begin{array}{c} \ln x=\int_{0}^{\infty }\frac{\cos(u)-\cos(ux)}{u}\;du. \end{array}$$

**Proof.**  By Proposition 1 and the identity lnx≡Reln(ix) (with $i:=\sqrt{-1}$), we get

(13) $$\begin{array}{cc} \ln x & =\int_{0}^{\infty }\frac{e^{-u}-\cos\left(ux\right)}{u}\;du. \end{array}$$

Subtracting both sides by the integral in (13) for x=1 (which is equal to zero) gives (12). □

Let *X* be a real-valued random variable, and let $\Phi_{X}\left(\nu \right):=E\left\{e^{i\nu X}\right\}$ be the characteristic function of *X*. Then, by Corollary 1,

(14) ElnX=∫0∞cos(u)−Re{ΦX(u)}udu,

where we are assuming, here and throughout the sequel, that the expectation operation and the integration over *u* are commutable, i.e., Fubini’s theorem applies.

Similarly, by returning to Proposition 1 (confined to a real-valued argument of the logarithm), the calculation of E{lnX} can be replaced by the calculation of the MGF of *X*, as

(15) $$\begin{array}{c} E\left\{\ln X\right\}=\int_{0}^{\infty }\left[e^{-u}-E\left\{e^{-uX}\right\}\right]\;\frac{du}{u}. \end{array}$$

In particular, if $X_{1},\ldots ,X_{n}$ are positive i.i.d. random variables, then

(16) $$\begin{array}{c} E\left\{\ln\left(X_{1}+\ldots +X_{n}\right)\right\}=\int_{0}^{\infty }\left[e^{-u}-{\left(E\left\{e^{-uX_{1}}\right\}\right)}^{n}\right]\;\frac{du}{u}. \end{array}$$

**Remark** **2.** *One may further manipulate (15) and (16) as follows. As $\ln x\equiv \frac{1}{s}\;\ln\left(x^{s}\right)$ for any s≠0 and x>0, then the expectation of lnX can also be represented as*

(17) $$\begin{array}{c} E\left\{\ln X\right\}=\frac{1}{s}\int_{0}^{\infty }\left[e^{-u}-E\left\{e^{-uX^{s}}\right\}\right]\;\frac{du}{u},\;s\neq 0. \end{array}$$

*The idea is that if, for some s∉{0,1}, $E\{e^{-uX^{s}}\}$ can be expressed in closed form, whereas it cannot for s=1 (or even $E\{e^{-uX^{s}}\}<\infty$ for some s∉{0,1}, but not for s=1), then (17) may prove useful. Moreover, if $X_{1},\ldots ,X_{n}$ are positive i.i.d. random variables, s>0, and $Y:={\left(X_{1}^{s}+\ldots +X_{n}^{s}\right)}^{1/s}$, then*

(18) $$\begin{array}{c} E\left\{\ln Y\right\}=\frac{1}{s}\int_{0}^{\infty }\left[e^{-u}-{\left(E\left\{e^{-uX_{1}^{s}}\right\}\right)}^{n}\right]\;\frac{du}{u}. \end{array}$$

*For example, if $\left\{X_{i}\right\}$ are i.i.d. standard Gaussian random variables and s=2, then (18) enables to calculate the expected value of the logarithm of a chi-squared distributed random variable with n degrees of freedom. In this case,*

(19) $$\begin{array}{ll} E\left\{e^{-uX_{1}^{2}}\right\} & =\frac{1}{\sqrt{2\pi }}\int_{-\infty }^{\infty }e^{-ux^{2}}\;e^{-x^{2}/2}\;dx\\ & =\frac{1}{\sqrt{2u+1}}, \end{array}$$

*and, from (18) with s=2,*

(20) $$\begin{array}{c} E\left\{\ln Y\right\}=\frac{1}{2}\int_{0}^{\infty }\left[e^{-u}-{\left(2u+1\right)}^{-n/2}\right]\;\frac{du}{u}. \end{array}$$

*Note that according to the *pdf* of a chi-squared distribution, one can express E{lnY} as a one-dimensional integral even without using (18). However, for general s>0, the direct calculation of $E\left\{\ln\left(\sum_{i=1}^{n}{\left|X_{i}\right|}^{s}\right)\right\}$ leads to an n-dimensional integral, whereas (18) provides a one-dimensional integral whose integrand involves in turn the calculation of a one-dimensional integral too.*

Identity (1) also proves useful when one is interested, not only in the expected value of lnX, but also in higher moments, in particular, its second moment or variance. In this case, the one-dimensional integral becomes a two-dimensional one. Specifically, for any s>0,

(21) $$\begin{array}{ll} Var\{\ln X\} & =E\left\{\ln^{2}\left(X\right)\right\}-{\left[E\left\{\ln X\right\}\right]}^{2}\\ \; & =\frac{1}{s^{2}}\;E\left\{\int_{0}^{\infty }\int_{0}^{\infty }\left(e^{-u}-e^{-uX^{s}}\right)\;\left(e^{-v}-e^{-vX^{s}}\right)\;\frac{du\;dv}{uv}\right\} \end{array}$$

(22) $$\begin{array}{cc} \; & \;-\frac{1}{s^{2}}\;\int_{0}^{\infty }\int_{0}^{\infty }\left(e^{-u}-E\left\{e^{-uX^{s}}\right\}\right)\;\left(e^{-v}-E\left\{e^{-vX^{s}}\right\}\right)\;\frac{du\;dv}{uv} \end{array}$$

(23) $$\begin{array}{cc} \; & =\frac{1}{s^{2}}\int_{0}^{\infty }\int_{0}^{\infty }\left[E\left\{e^{-\left(u+v\right)X^{s}}\right\}-E\left\{e^{-uX^{s}}\right\}\;E\left\{e^{-vX^{s}}\right\}\right]\;\frac{du\;dv}{uv} \end{array}$$

(24) $$\begin{array}{cc} \; & =\frac{1}{s^{2}}\int_{0}^{\infty }\int_{0}^{\infty }\mathrm{Cov}\left\{e^{-uX^{s}},e^{-vX^{s}}\right\}\;\frac{du\;dv}{uv}. \end{array}$$

More generally, for a pair of positive random variables, (X,Y), and for s>0,

(25) $$\begin{array}{c} \mathrm{Cov}\left\{\ln X,\ln Y\right\}=\frac{1}{s^{2}}\int_{0}^{\infty }\int_{0}^{\infty }\mathrm{Cov}\left\{e^{-uX^{s}},e^{-vY^{s}}\right\}\;\frac{du\;dv}{uv}. \end{array}$$

For later use, we present the following variation of the basic identity.

**Proposition** **2.** *Let X be a random variable, and let*

(26) $$\begin{array}{c} M_{X}\left(s\right):=E\left\{e^{sX}\right\},\;\forall \;s\in R, \end{array}$$

*be the MGF of X. If X is non-negative, then*

(27) $$\begin{array}{cc} \; & E\left\{\ln(1+X)\right\}=\int_{0}^{\infty }\frac{e^{-u}\;\left[1-M_{X}\left(-u\right)\right]}{u}\;du, \end{array}$$

(28) $$\begin{array}{cc} \; & \mathrm{Var}\left\{\ln(1+X)\right\}=\int_{0}^{\infty }\int_{0}^{\infty }\frac{e^{-(u+v)}}{uv}\;\left[M_{X}\left(-u-v\right)-M_{X}\left(-u\right)\;M_{X}\left(-v\right)\right]\;du\;dv. \end{array}$$

**Proof.**  Equation (27) is a trivial consequence of (15). As for (28), we have

(29) $$\begin{array}{ll} \mathrm{Var}\left\{\ln(1+X)\right\} & =E\left\{\ln^{2}\left(1+X\right)\right\}-{\left(E\left\{\ln(1+X)\right\}\right)}^{2}\\ \; & =E\left\{\int_{0}^{\infty }\frac{e^{-u}}{u}\;\left(1-e^{-uX}\right)\;du\int_{0}^{\infty }\frac{e^{-v}}{v}\;\left(1-e^{-vX}\right)\;dv\right\}\\ \; & \;-\int_{0}^{\infty }\int_{0}^{\infty }\frac{e^{-(u+v)}\;\left[1-M_{X}\left(-u\right)\right]\;\left[1-M_{X}\left(-v\right)\right]}{uv}\;du\;dv\\ \; & =\int_{0}^{\infty }\int_{0}^{\infty }\frac{e^{-(u+v)}}{uv}\;E\left\{\left(1-e^{-uX}\right)\;\left(1-e^{-vX}\right)\right\}\;du\;dv \end{array}$$

(30) $$\begin{array}{cc} \; & \;-\int_{0}^{\infty }\int_{0}^{\infty }\frac{e^{-(u+v)}\;\left[1-M_{X}\left(-u\right)-M_{X}\left(-v\right)+M_{X}\left(-u\right)\;M_{X}\left(-v\right)\right]}{uv}\;du\;dv\\ \; & =\int_{0}^{\infty }\int_{0}^{\infty }\frac{e^{-(u+v)}}{uv}\;\left[1-M_{X}\left(-u\right)-M_{X}\left(-v\right)+M_{X}\left(-u-v\right)\right]\;du\;dv \end{array}$$

(31) $$\begin{array}{cc} \; & \;-\int_{0}^{\infty }\int_{0}^{\infty }\frac{e^{-(u+v)}}{uv}\;\left[1-M_{X}\left(-u\right)-M_{X}\left(-v\right)+M_{X}\left(-u\right)\;M_{X}\left(-v\right)\right]\;du\;dv \end{array}$$

(32) $$\begin{array}{cc} \; & =\int_{0}^{\infty }\int_{0}^{\infty }\frac{e^{-(u+v)}}{uv}\;\left[M_{X}\left(-u-v\right)-M_{X}\left(-u\right)\;M_{X}\left(-v\right)\right]du\;dv. \end{array}$$

 □

The following result relies on the validity of (5) to the right-half complex plane, and its derivation is based on the identity $\ln\left(1+x^{2}\right)\equiv \ln\left(1+ix\right)+\ln\left(1-ix\right)$ for all x∈R. In general, it may be used if the characteristic function of a random variable *X* has a closed-form expression, whereas the MGF of $X^{2}$ does not admit a closed-form expression (see Proposition 2). We introduce the result, although it is not directly used in the paper.

**Proposition** **3.** *Let X be a real-valued random variable, and let*

(33) $$\begin{array}{c} \Phi_{X}\left(u\right):=E\left\{e^{iuX}\right\},\;\forall \;u\in R, \end{array}$$

*be the characteristic function of X. Then,*

(34) Eln(1+X2)=2∫0∞e−uu1−ReΦX(u)du,

*and*

(35) Varln(1+X2)=2∫0∞∫0∞e−u−vuv[Re{ΦX(u+v)}+Re{ΦX(u−v)}−2Re{ΦX(u)}Re{ΦX(v)}]dudv.

As a final note, we point out that the fact that the integral representation (2) replaces the expectation of the logarithm of *X* by the expectation of an exponential function of *X*, has an additional interesting consequence: an expression like ln(n!) becomes the integral of the sum of a geometric series, which, in turn, is easy to express in closed form (see [11] ((2.3)–(2.4))). Specifically,

(36) $$\begin{array}{ll} \ln(n!) & =\sum_{k=1}^{n}\ln k\\ & =\sum_{k=1}^{n}\int_{0}^{\infty }\left(e^{-u}-e^{-uk}\right)\;\frac{du}{u}\\ & =\int_{0}^{\infty }\left(ne^{-u}-\sum_{k=1}^{n}e^{-uk}\right)\frac{du}{u}\\ & =\int_{0}^{\infty }e^{-u}\left(n-\frac{1-e^{-un}}{1-e^{-u}}\right)\frac{du}{u}. \end{array}$$

Thus, for a positive integer-valued random variable, *N*, the calculation of E{lnN!} requires merely the calculation of E{N} and the MGF, $E\{e^{-uN}\}$. For example, if *N* is a Poissonian random variable, as discussed near the end of the Introduction, both E{N} and $E\{e^{-uN}\}$ are easy to evaluate. This approach is a simple, direct alternative to the one taken in [8] (see also [10]), where Malmstén’s nontrivial formula for lnΓ(z) (see [9] (pp. 20–21)) was invoked. (Malmstén’s formula for lnΓ(z) applies to a general, complex–valued *z* with Re(z)>0; in the present context, however, only integer real values of *z* are needed, and this allows the simplification shown in (36)). The above described idea of the geometric series will also be used in one of our application examples, in Section 3.4.

## 3. Applications

In this section, we show the usefulness of the integral representation of the logarithmic function in several problem areas in information theory. To demonstrate the direct computability of the relevant quantities, we also present graphs of their numerical calculation. In some of the examples, we also demonstrate calculations of the second moments and variances.

### 3.1. Differential Entropy for Generalized Multivariate Cauchy Densities

Let $\left(X_{1},\ldots ,X_{n}\right)$ be a random vector whose probability density function is of the form

(37) $$\begin{array}{c} f\left(x_{1},\ldots ,x_{n}\right)=\frac{C_{n}}{{\left[1+\sum_{i=1}^{n}g\left(x_{i}\right)\right]}^{q}},\;\forall \;\left(x_{1},\ldots ,x_{n}\right)\in R^{n}, \end{array}$$

for a certain non–negative function *g* and positive constant *q* such that

(38) $$\begin{array}{c} \int_{R^{n}}\frac{dx}{{\left[1+\sum_{i=1}^{n}g\left(x_{i}\right)\right]}^{q}}<\infty . \end{array}$$

We refer to this kind of density as a generalized multivariate Cauchy density, because the multivariate Cauchy density is obtained as a special case where $g\left(x\right)=x^{2}$ and $q=\frac{1}{2}\left(n+1\right)$. Using the Laplace transform relation,

(39) 1sq=1Γ(q)∫0∞tq−1e−stdt,∀q>0,Re(s)>0,

*f* can be represented as a mixture of product measures:

(40) $$\begin{array}{ll} f(x_{1},\ldots ,x_{n}) & =\frac{C_{n}}{{\left[1+\sum_{i=1}^{n}g\left(x_{i}\right)\right]}^{q}}\\ & =\frac{C_{n}}{\Gamma (q)}\int_{0}^{\infty }t^{q-1}e^{-t}\;\exp\left\{-t\sum_{i=1}^{n}g\left(x_{i}\right)\right\}\;dt. \end{array}$$

Defining

(41) $$\begin{array}{c} Z\left(t\right):=\int_{-\infty }^{\infty }e^{-tg(x)}\;dx,\;\forall \;t>0, \end{array}$$

we get from (40),

(42) $$\begin{array}{ll} 1 & =\frac{C_{n}}{\Gamma (q)}\int_{0}^{\infty }t^{q-1}e^{-t}\int_{R^{n}}\exp\left\{-t\sum_{i=1}^{n}g\left(x_{i}\right)\right\}\;dx_{1}\;\ldots \;dx_{n}\;dt\\ & =\frac{C_{n}}{\Gamma (q)}\int_{0}^{\infty }t^{q-1}e^{-t}{\left(\int_{-\infty }^{\infty }e^{-tg(x)}\;dx\right)}^{n}dt\\ & =\frac{C_{n}}{\Gamma (q)}\int_{0}^{\infty }t^{q-1}e^{-t}Z^{n}\left(t\right)\;dt, \end{array}$$

and so,

(43) $$\begin{array}{c} C_{n}=\frac{\Gamma (q)}{\int_{0}^{\infty }t^{q-1}e^{-t}Z^{n}\left(t\right)\;dt}. \end{array}$$

The calculation of the differential entropy of *f* is associated with the evaluation of the expectation $E\left\{\ln\left[1+\sum_{i=1}^{n}g\left(X_{i}\right)\right]\right\}$. Using (27),

(44) $$\begin{array}{c} E\left\{\ln\left[1+\sum_{i=1}^{n}g\left(X_{i}\right)\right]\right\}=\int_{0}^{\infty }\frac{e^{-u}}{u}\left(1-E\left\{\exp\left[-u\sum_{i=1}^{n}g\left(X_{i}\right)\right]\right\}\right)du. \end{array}$$

From (40) and by interchanging the integration,

(45) $$\begin{array}{ll} E\left\{\exp\left[-u\sum_{i=1}^{n}g\left(X_{i}\right)\right]\right\}\; & =\frac{C_{n}}{\Gamma (q)}\int_{0}^{\infty }t^{q-1}e^{-t}\int_{R^{n}}\exp\left\{-\left(t+u\right)\sum_{i=1}^{n}g\left(x_{i}\right)\right\}dx_{1}\;\ldots dx_{n}\;dt\\ & =\frac{C_{n}}{\Gamma (q)}\int_{0}^{\infty }t^{q-1}e^{-t}Z^{n}\left(t+u\right)\;dt. \end{array}$$

In view of (40), (44), and (45), the differential entropy of $\left(X_{1},\ldots ,X_{n}\right)$ is therefore given by

(46) $$\begin{array}{ll} h(X_{1},\ldots ,X_{n}) & =q\;E\left\{\ln\left[1+\sum_{i=1}^{n}g\left(X_{i}\right)\right]\right\}-\ln C_{n}\\ & =q\int_{0}^{\infty }\frac{e^{-u}}{u}\left(1-\frac{C_{n}}{\Gamma (q)}\int_{0}^{\infty }t^{q-1}e^{-t}Z^{n}\left(t+u\right)dt\right)du-\ln C_{n}\\ & =\frac{qC_{n}}{\Gamma (q)}\int_{0}^{\infty }\int_{0}^{\infty }\frac{t^{q-1}e^{-(t+u)}}{u}\;\left[Z^{n}\left(t\right)-Z^{n}\left(t+u\right)\right]\;dt\;du-\ln C_{n}. \end{array}$$

For $g\left(x\right)={\left|x\right|}^{\theta }$, with an arbitrary θ>0, we obtain from (41) that

(47) $$\begin{array}{c} Z\left(t\right)=\frac{2\;\Gamma (1/\theta )}{\theta \;t^{1/\theta }}. \end{array}$$

In particular, for θ=2 and $q=\frac{1}{2}\left(n+1\right)$, we get the multivariate Cauchy density from (37). In this case, as $\Gamma \left(\frac{1}{2}\right)=\sqrt{\pi }$, it follows from (47) that $Z\left(t\right)=\sqrt{\frac{\pi }{t}}$ for t>0, and from (43)

(48) $$\begin{array}{ll} C_{n} & =\frac{\Gamma \left(\frac{n+1}{2}\right)}{\pi^{n/2}\;\int_{0}^{\infty }t^{(n+1)/2-1}e^{-t}\;t^{-n/2}\;dt}\\ & =\frac{\Gamma \left(\frac{n+1}{2}\right)}{\pi^{n/2}\;\Gamma \left(\frac{1}{2}\right)}\\ & =\frac{\Gamma \left(\frac{n+1}{2}\right)}{\pi^{(n+1)/2}}. \end{array}$$

Combining (46), (47) and (48) gives

(49) $$\begin{array}{ll} h(X_{1},\ldots ,X_{n}) & =\frac{n+1}{2\pi^{(n+1)/2}}\int_{0}^{\infty }\int_{0}^{\infty }\frac{e^{-(t+u)}}{u\sqrt{t}}\left[1-{\left(\frac{t}{t+u}\right)}^{n/2}\right]\;dt\;du\\ & +\frac{(n+1)\ln\pi }{2}-\ln\Gamma \left(\frac{n+1}{2}\right). \end{array}$$

**Figure 1:**
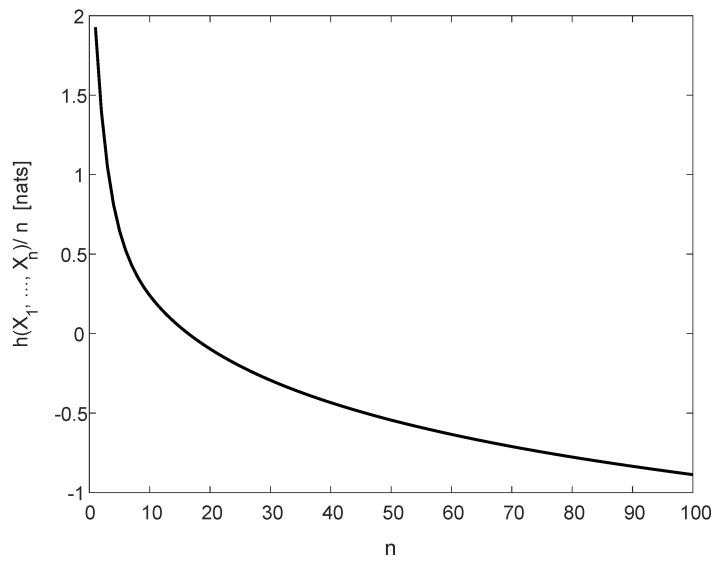
The normalized differential entropy, $\frac{1}{n}\;h\left(X_{1},\ldots ,X_{n}\right)$ (see (49)), for a multivariate Cauchy density, $f\left(x_{1},\ldots ,x_{n}\right)=C_{n}/{\left[1+\sum_{i=1}^{n}x_{i}^{2}\right]}^{(n+1)/2}$, with $C_{n}$ in (48).

Figure 1 displays the normalized differential entropy, $\frac{1}{n}\;h\left(X_{1},\ldots ,X_{n}\right)$, for 1≤n≤100.

We believe that the interesting point, conveyed in this application example, is that (46) provides a kind of a “single–letter expression”; the *n*–dimensional integral, associated with the original expression of the differential entropy $h(X_{1},\ldots ,X_{n})$, is replaced by the two-dimensional integral in (46), independently of *n*.

As a final note, we mention that a lower bound on the differential entropy of a different form of extended multivariate Cauchy distributions (cf. [13] (Equation (42))) was derived in [13] (Theorem 6). The latter result relies on obtaining lower bounds on the differential entropy of random vectors whose densities are symmetric log-concave or γ-concave (i.e., densities *f* for which $f^{\gamma }$ is concave for some γ<0).

### 3.2. Ergodic Capacity of the Fading SIMO Channel

Consider the SIMO channel with *L* receive antennas and assume that the channel transfer coefficients, ${\left\{h_{i}\right\}}_{i=1}^{L}$, are independent, zero–mean, circularly symmetric complex Gaussian random variables with variances ${\left\{\sigma_{i}^{2}\right\}}_{i=1}^{L}$. Its ergodic capacity (in nats per channel use) is given by

(50) $$\begin{array}{c} C=E\left\{\ln\left(1+\rho \sum_{\ell =1}^{L}{\left|h_{\ell }\right|}^{2}\right)\right\}=E\left\{\ln\left(1+\rho \sum_{\ell =1}^{L}\left(f_{\ell }^{2}+g_{\ell }^{2}\right)\right)\right\}, \end{array}$$

where fℓ:=Re{hℓ}, gℓ:=Im{hℓ}, and $\rho :=\frac{P}{N_{0}}$ is the signal–to–noise ratio (SNR) (see, e.g., [14,15]).

Paper [14] is devoted, among other things, to the exact evaluation of (50) by finding the density of the random variable defined by $\sum_{\ell =1}^{L}\left(f_{\ell }^{2}+g_{\ell }^{2}\right)$, and then taking the expectation w.r.t. that density. Here, we show that the integral representation in (5) suggests a more direct approach to the evaluation of (50). It should also be pointed out that this approach is more flexible than the one in [14], as the latter strongly depends on the assumption that $\left\{h_{i}\right\}$ are Gaussian and statistically independent. The integral representation approach also allows other distributions of the channel transfer gains, as well as possible correlations between the coefficients and/or the channel inputs. Moreover, we are also able to calculate the variance of $\ln\left(1+\rho \sum_{\ell =1}^{L}{\left|h_{\ell }\right|}^{2}\right)$, as a measure of the fluctuations around the mean, which is obviously related to the outage.

Specifically, in view of Proposition 2 (see (27)), let

(51) $$\begin{array}{c} X:=\rho \;\sum_{\ell =1}^{L}\left(f_{\ell }^{2}+g_{\ell }^{2}\right). \end{array}$$

For all u>0,

(52) $$\begin{array}{ll} M_{X}\left(-u\right) & =E\left\{\exp\left(-\rho u\;\sum_{\ell =1}^{L}\left(f_{\ell }^{2}+g_{\ell }^{2}\right)\right)\right\}\\ & =\prod_{\ell =1}^{L}\left\{E\left\{e^{-u\rho f_{\ell }^{2}}\right\}\;E\left\{e^{-u\rho g_{\ell }^{2}}\right\}\right\}\\ & =\prod_{\ell =1}^{L}\frac{1}{1+u\rho \sigma_{\ell }^{2}}, \end{array}$$

where (52) holds since

(53) $$\begin{array}{ll} E\left\{e^{-u\rho f_{\ell }^{2}}\right\} & =E\left\{e^{-u\rho g_{\ell }^{2}}\right\}\\ & =\int_{-\infty }^{\infty }\frac{dw}{\sqrt{\pi \sigma_{\ell }^{2}}}\;e^{-w^{2}/\sigma_{\ell }^{2}}\;e^{-u\rho w^{2}}\\ & =\frac{1}{\sqrt{1+u\rho \sigma_{\ell }^{2}}}. \end{array}$$

From (27), (50) and (52), the ergodic capacity (in nats per channel use) is given by

(54) $$\begin{array}{ll} C & =E\left\{\ln\left(1+\rho \sum_{\ell =1}^{L}\left(f_{\ell }^{2}+g_{\ell }^{2}\right)\right)\right\}\\ & =\int_{0}^{\infty }\frac{e^{-u}}{u}\left(1-\prod_{\ell =1}^{L}\frac{1}{1+u\rho \sigma_{\ell }^{2}}\right)\;du\\ & =\int_{0}^{\infty }\frac{e^{-x/\rho }}{x}\left(1-\prod_{\ell =1}^{L}\frac{1}{1+\sigma_{\ell }^{2}x}\right)\;dx. \end{array}$$

A similar approach appears in [4] (Equation (12)).

As for the variance, from Proposition 2 (see (28)) and (52),

(55) $$\begin{array}{ll} \; & \mathrm{Var}\left\{\ln\left(1+\rho \sum_{\ell =1}^{L}\left[f_{\ell }^{2}+g_{\ell }^{2}\right]\right)\right\}\\ \; & =\int_{0}^{\infty }\int_{0}^{\infty }\frac{e^{-(x+y)/\rho }}{xy}\;\left\{\prod_{\ell =1}^{L}\frac{1}{1+\sigma_{\ell }^{2}\left(x+y\right)}-\prod_{\ell =1}^{L}\left[\frac{1}{\left(1+\sigma_{\ell }^{2}x\right)\left(1+\sigma_{\ell }^{2}y\right)}\right]\right\}\;dx\;dy. \end{array}$$

A similar analysis holds for the multiple-input single-output (MISO) channel. By partial–fraction decomposition of the expression (see the right side of (54))

$$\frac{1}{x}\left(1-\prod_{\ell =1}^{L}\frac{1}{1+\sigma_{\ell }^{2}x}\right),$$

the ergodic capacity *C* can be expressed as a linear combination of integrals of the form

(56) $$\begin{array}{ll} \int_{0}^{\infty }\frac{e^{-x/\rho }\;dx}{1+\sigma_{\ell }^{2}x} & =\frac{1}{\sigma_{\ell }^{2}}\int_{0}^{\infty }\frac{e^{-t}\;dt}{t+1/(\sigma_{\ell }^{2}\rho )}\\ & =\frac{e^{1/(\sigma_{\ell }^{2}\rho )}}{\sigma_{\ell }^{2}}\int_{1/(\sigma_{\ell }^{2}\rho )}^{\infty }\frac{e^{-s}}{s}\;ds\\ & =\frac{1}{\sigma_{\ell }^{2}}\;e^{1/(\sigma_{\ell }^{2}\rho )}\;E_{1}\left(\frac{1}{\sigma_{\ell }^{2}\rho }\right), \end{array}$$

where $E_{1}\left(\cdot \right)$ is the (modified) exponential integral function, defined as

(57) $$\begin{array}{c} E_{1}\left(x\right):=\int_{x}^{\infty }\frac{e^{-s}}{s}\;ds,\;\forall \;x>0. \end{array}$$

A similar representation appears also in [14] (Equation (7)).

Consider the example of L=2, $\sigma_{1}^{2}=\frac{1}{2}$ and $\sigma_{2}^{2}=1$. From (54), the ergodic capacity of the SIMO channel is given by

(58) $$\begin{array}{ll} C & =\int_{0}^{\infty }\frac{e^{-x/\rho }}{x}\left[1-\frac{1}{\left(x/2+1)(x+1\right)}\right]dx\\ & =\int_{0}^{\infty }\frac{e^{-x/\rho }\;\left(x+3\right)\;dx}{\left(x+1)(x+2\right)}\\ & =2e^{1/\rho }\;E_{1}\left(\frac{1}{\rho }\right)-e^{2/\rho }\;E_{1}\left(\frac{2}{\rho }\right). \end{array}$$

The variance in this example (see (55)) is given by

(59) $$\begin{array}{ll} \; & \mathrm{Var}\left\{\ln\left(1+\rho \sum_{\ell =1}^{2}\left(f_{\ell }^{2}+g_{\ell }^{2}\right)\right)\right\}\\ \; & =\int_{0}^{\infty }\int_{0}^{\infty }\frac{e^{-(x+y)/\rho }}{xy}\;\left[\frac{1}{\left(1+0.5(x+y)\right)\left(1+x+y\right)}-\frac{1}{\left(1+0.5x)(1+0.5y)(1+x)(1+y\right)}\right]\;dx\;dy\\ \; & =\int_{0}^{\infty }\int_{0}^{\infty }\frac{e^{-(x+y)/\rho }\;\left(2xy+6x+6y+10\right)\;dx\;dy}{\left(x+1)(y+1)(x+2)(y+2)(x+y+1)(x+y+2\right)}. \end{array}$$

**Figure 2:**
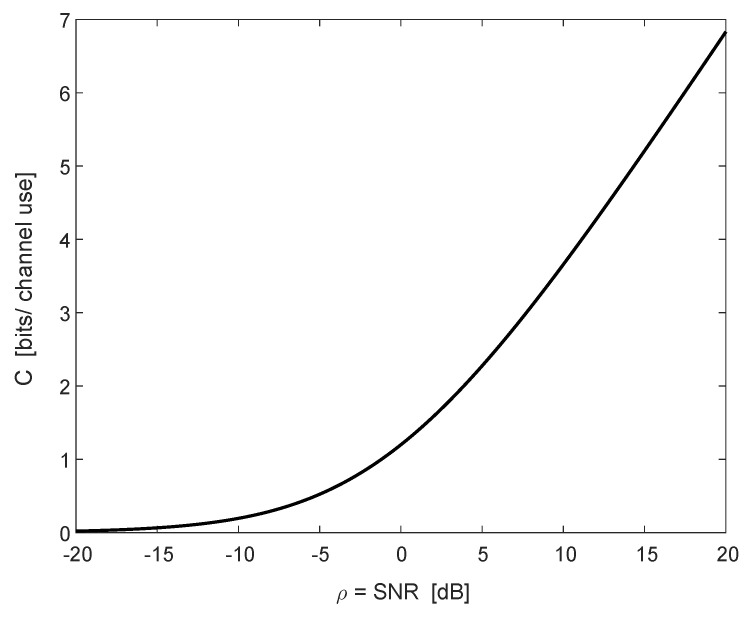
The ergodic capacity *C* (in bits per channel use) of the SIMO channel as a function of ρ=SNR (in dB) for L=2 receive antennas, with noise variances $\sigma_{1}^{2}=\frac{1}{2}$ and $\sigma_{2}^{2}=1$.

**Figure 3:**
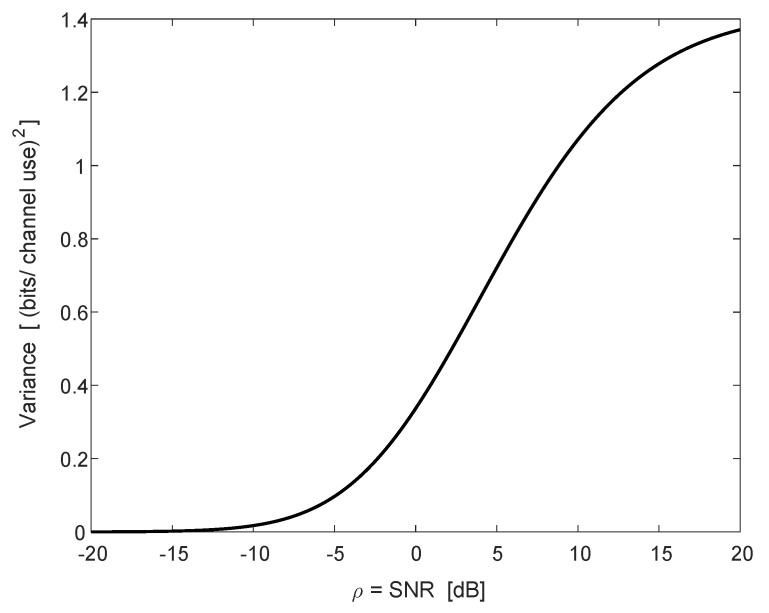
The variance of $\ln(1+\rho \sum_{\ell =1}^{L}|h_{\ell }{|}^{2})$ (in ${\left[\mathrm{bits}-\mathrm{per}-\mathrm{channel}-\mathrm{use}\right]}^{2}$) of the SIMO channel as a function of ρ=SNR (in dB) for L=2 receive antennas, with noise variances $\sigma_{1}^{2}=\frac{1}{2}$ and $\sigma_{2}^{2}=1$.

Figure 2 depicts the ergodic capacity *C* as a function of the SNR, ρ, in dB (see (58), and divide by ln2 for conversion to bits per channel use). The same example exactly appears in the lower graph of Figure 1 in [14]. The variance appears in Figure 3 (see (59), and similarly divide by $\ln^{2}2$).

### 3.3. Universal Source Coding for Binary Arbitrarily Varying Sources

Consider a source coding setting, where there are *n* binary DMS’s, and let $x_{i}\in \left[0,1\right]$ denote the Bernoulli parameter of source no. i∈{1,…,n}. Assume that a hidden memoryless switch selects uniformly at random one of these sources, and the data is then emitted by the selected source. Since it is unknown a-priori which source is selected at each instant, a universal lossless source encoder (e.g., a Shannon or Huffman code) is designed to match a binary DMS whose Bernoulli parameter is given by $\frac{1}{n}\sum_{i=1}^{n}x_{i}$. Neglecting integer length constraints, the average redundancy in the compression rate (measured in nats per symbol), due to the unknown realization of the hidden switch, is about

(60) $$\begin{array}{c} R_{n}=h_{b}\left(\frac{1}{n}\sum_{i=1}^{n}x_{i}\right)-\frac{1}{n}\sum_{i=1}^{n}h_{b}\left(x_{i}\right), \end{array}$$

where $h_{b}:\left[0,1\right]\to \left[0,\ln2\right]$ is the binary entropy function (defined to the base *e*), and the redundancy is given in nats per source symbol. Now, let us assume that the Bernoulli parameters of the *n* sources are i.i.d. random variables, $X_{1},\ldots ,X_{n}$, all having the same density as that of some generic random variable *X*, whose support is the interval [0,1]. We wish to evaluate the expected value of the above defined redundancy, under the assumption that the realizations of $X_{1},\ldots ,X_{n}$ are known. We are then facing the need to evaluate

(61) $$\begin{array}{c} {\bar{R}}_{n}=E\left\{h_{b}\left(\frac{1}{n}\sum_{i=1}^{n}X_{i}\right)\right\}-E\left\{h_{b}\left(X\right)\right\}. \end{array}$$

We now express the first and second terms on the right-hand side of (61) as a function of the MGF of *X*.

In view of (5), the binary entropy function $h_{b}$ admits the integral representation

(62) $$\begin{array}{c} h_{b}\left(x\right)=\int_{0}^{\infty }\frac{1}{u}\;\left[xe^{-ux}+\left(1-x\right)e^{-u(1-x)}-e^{-u}\right]\;du,\;\forall \;x\in \left[0,1\right], \end{array}$$

which implies that

(63) $$\begin{array}{cc} E\{h_{b}\left(X\right)\} & =\int_{0}^{\infty }\frac{1}{u}\;\left[E\left\{Xe^{-uX}\right\}+E\left\{\left(1-X\right)e^{-u(1-X)}\right\}-e^{-u}\right]\;du. \end{array}$$

The expectations on the right-hand side of (63) can be expressed as functionals of the MGF of *X*, $M_{X}\left(\nu \right)=E\left\{e^{\nu X}\right\}$, and its derivative, for ν<0. For all u∈R,

(64) $$\begin{array}{cc} \; & E\left\{Xe^{-uX}\right\}=M_{X}^{\prime }\left(-u\right), \end{array}$$

and

(65) $$\begin{array}{ll} E\left\{\left(1-X\right)e^{-u(1-X)}\right\} & =M_{1-X}^{\prime }\left(-u\right)\\ & =\frac{d}{ds}\left\{e^{s}\;M_{X}\left(-s\right)\right\}{|}_{s=-u}\\ & =e^{-u}\left[M_{X}\left(u\right)-M_{X}^{\prime }\left(u\right)\right]. \end{array}$$

On substituting (64) and (65) into (63), we readily obtain

(66) $$\begin{array}{cc} E\{h_{b}\left(X\right)\} & =\int_{0}^{\infty }\frac{1}{u}\;\left\{M_{X}^{\prime }\left(-u\right)+\left[M_{X}\left(u\right)-M_{X}^{\prime }\left(u\right)-1\right]e^{-u}\right\}\;du. \end{array}$$

Define $Y_{n}:=\frac{1}{n}\sum_{i=1}^{n}X_{i}$. Then,

(67) $$\begin{array}{c} M_{Y_{n}}\left(u\right)=M_{X}^{n}\left(\frac{u}{n}\right),\;\forall \;u\in R, \end{array}$$

which yields, in view of (66), (67) and the change of integration variable, $t=\frac{u}{n}$, the following:

(68) $$\begin{array}{ll} E\left\{h_{b}\left(\frac{1}{n}\sum_{i=1}^{n}X_{i}\right)\right\} & =E\{h_{b}\left(Y_{n}\right)\}\\ & =\int_{0}^{\infty }\frac{1}{u}\;\left\{M_{Y_{n}}^{\prime }\left(-u\right)+\left[M_{Y_{n}}\left(u\right)-M_{Y_{n}}^{\prime }\left(u\right)-1\right]e^{-u}\right\}\;du\\ & =\int_{0}^{\infty }\frac{1}{t}\;\left\{M_{X}^{n-1}\left(-t\right)\;M_{X}^{\prime }\left(-t\right)+\left[M_{X}^{n}\left(t\right)-M_{X}^{n-1}\left(t\right)\;M_{X}^{\prime }\left(t\right)-1\right]\;e^{-nt}\right\}\;dt. \end{array}$$

Similarly as in Section 3.1, here too, we pass from an *n*-dimensional integral to a one-dimensional integral. In general, similar calculations can be carried out for higher integer moments, thus passing from *n*-dimensional integration for a moment of order *s* to an *s*-dimensional integral, independently of *n*.

For example, if $X_{1},\ldots ,X_{n}$ are i.i.d. and uniformly distributed on [0,1], then the MGF of a generic random variable *X* distributed like all $\left\{X_{i}\right\}$ is given by

(69) $$M_{X}\left(t\right)=\{\begin{array}{cc} \frac{e^{t}-1}{t},\; & t\neq 0,\\ 1,\; & t=0. \end{array}$$

From (68), it can be verified numerically that $E\left\{h_{b}\left(\frac{1}{n}\sum_{i=1}^{n}X_{i}\right)\right\}$ is monotonically increasing in *n*, being equal (in nats) to $\frac{1}{2}$, 0.602, 0.634, 0.650, 0.659 for n=1,…,5, respectively, with the limit $h_{b}\left(\frac{1}{2}\right)=\ln2\approx 0.693$ as we let n→∞ (this is expected by the law of large numbers).

### 3.4. Moments of the Empirical Entropy and the Redundancy of K–T Universal Source Coding

Consider a stationary, discrete memoryless source (DMS), *P*, with a finite alphabet X of size |X| and letter probabilities {P(x),x∈X}. Let $\left(X_{1},\ldots ,X_{n}\right)$ be an *n*–vector emitted from *P*, and let $\left\{\hat{P}\left(x\right),\;x\in X\right\}$ be the empirical distribution associated with $\left(X_{1},\ldots ,X_{n}\right)$, that is, $\hat{P}\left(x\right)=\frac{n(x)}{n}$, for all x∈X, where n(x) is the number of occurrences of the letter *x* in $\left(X_{1},\ldots ,X_{n}\right)$.

It is well known that in many universal lossless source codes for the class of memoryless sources, the dominant term of the length function for encoding $\left(X_{1},\ldots ,X_{n}\right)$ is $n\hat{H}$, where $\hat{H}$ is the empirical entropy,

(70) $$\begin{array}{c} \hat{H}=-\sum_{x}\hat{P}\left(x\right)\ln\hat{P}\left(x\right). \end{array}$$

For code length performance analysis (as well as for entropy estimation per se), there is therefore interest in calculating the expected value $E\{\hat{H}\}$ as well as $\mathrm{Var}\{\hat{H}\}$. Another motivation comes from the quest for estimating the entropy as an objective on its own right, and then the expectation and the variance suffice for the calculation of the mean square error of the estimate, $\hat{H}$. Most of the results that are available in the literature, in this context, concern the asymptotic behavior for large *n* as well as bounds (see, e.g., [16,17,18,19,20,21,22,23,24,25,26,27,28,29,30], as well as many other related references therein). The integral representation of the logarithm in (5), on the other hand, allows exact calculations of the expectation and the variance. The expected value of the empirical entropy is given by

(71) $$\begin{array}{ll} E\{\hat{H}\} & =-\sum_{x}E\left\{\hat{P}\left(x\right)\ln\hat{P}\left(x\right)\right\}\\ & =\sum_{x}E\left\{\int_{0}^{\infty }\frac{du}{u}\left[\hat{P}\left(x\right)e^{-u\hat{P}\left(x\right)}-\hat{P}\left(x\right)e^{-u}\right]\right\}\\ & =\int_{0}^{\infty }\frac{du}{u}\left[\sum_{x}E\left\{\hat{P}\left(x\right)e^{-u\hat{P}\left(x\right)}\right\}-e^{-u}\right]. \end{array}$$

For convenience, let us define the function $\phi_{n}:X\times R\to \left(0,\infty \right)$ as

(72) $$\begin{array}{c} \phi_{n}\left(x,t\right):=E\left\{e^{t\hat{P}\left(x\right)}\right\}={\left[1-P\left(x\right)+P\left(x\right)e^{t/n}\right]}^{n}, \end{array}$$

which yields,

(73) $$\begin{array}{cc} \; & E\left\{\hat{P}\left(x\right)e^{-u\hat{P}\left(x\right)}\right\}=\phi_{n}^{\prime }\left(x,-u\right), \end{array}$$

(74) $$\begin{array}{cc} \; & E\left\{{\hat{P}}^{2}\left(x\right)e^{-u\hat{P}\left(x\right)}\right\}=\phi_{n}^{\prime \prime }\left(x,-u\right), \end{array}$$

where $\phi_{n}^{\prime }$ and $\phi_{n}^{\prime \prime }$ are first and second order derivatives of $\phi_{n}$ w.r.t. *t*, respectively. From (71) and (73),

(75) $$\begin{array}{ll} E\{\hat{H}\}\; & =\int_{0}^{\infty }\frac{du}{u}\left(\sum_{x}\phi_{n}^{\prime }\left(x,-u\right)-e^{-u}\right)\\ & =\int_{0}^{\infty }\frac{du}{u}\left(e^{-u}\sum_{x}P\left(x\right){\left[1-P\left(x\right)\left(1-e^{-u}\right)\right]}^{n-1}-e^{-nu}\right) \end{array}$$

where the integration variable in (75) was changed using a simple scaling by *n*.

Before proceeding with the calculation of the variance of $\hat{H}$, let us first compare the integral representation in (75) to the alternative sum, obtained by a direct, straightforward calculation of the expected value of the empirical entropy. A straightforward calculation gives

(76) E{H^}=∑x∑k=0nnkPk(x)[1−P(x)]n−k·kn·lnnk

(77) =∑x∑k=1nn−1k−1Pk(x)[1−P(x)]n−k·lnnk.

We next compare the computational complexity of implementing (75) to that of (77). For large *n*, in order to avoid numerical problems in computing (77) by standard software, one may use the Gammaln function in Matlab/Excel or the LogGamma in Mathematica (a built-in function for calculating the natural logarithm of the Gamma function) to obtain that

(78) n−1k−1Pk(x)[1−P(x)]n−k=exp{Gammaln(n)−Gammaln(k)−Gammaln(n−k+1)+klnP(x)+(n−k)ln1−P(x)}.

The right-hand side of (75) is the sum of |X| integrals, and the computational complexity of each integral depends on neither *n*, nor |X|. Hence, the computational complexity of the right-hand side of (75) scales *linearly* with |X|. On the other hand, the double sum on the right-hand side of (77) consists of n·|X| terms. Let $\alpha :=\frac{n}{\left|X\right|}$ be fixed, which is expected to be large (α≫1) if a good estimate of the entropy is sought. The computational complexity of the double sum on the right-hand side of (77) grows like ${\alpha \;|X|}^{2}$, which scales *quadratically* in |X|. Hence, for a DMS with a large alphabet, or when n≫|X|, there is a significant computational reduction by evaluating (75) in comparison to the right-hand side of (77).

We next move on to calculate the variance of $\hat{H}$.

(79) $$\begin{array}{ll} \mathrm{Var}\{\hat{H}\} & =E\left\{{\hat{H}}^{2}\right\}-E^{2}\left\{\hat{H}\right\} \end{array}$$

(80) $$\begin{array}{cc} \; & =\sum_{x,x^{\prime }}E\left\{\hat{P}\left(x\right)\ln\hat{P}\left(x\right)\cdot \hat{P}\left(x^{\prime }\right)\ln\hat{P}\left(x^{\prime }\right)\right\}-E^{2}\left\{\hat{H}\right\}. \end{array}$$

The second term on the right-hand side of (80) has already been calculated. For the first term, let us define, for $x^{\prime }\neq x$,

(81) $$\begin{array}{ll} \psi_{n}\left(x,x^{\prime },s,t\right) & :=E\{\exp\left\{s\hat{P}\left(x\right)+t\hat{P}\left(x^{\prime }\right)\right\}\\ \; & \;=\sum_{\left\{(k,\ell ):\;k+\ell \leq n\right\}}\{\frac{n!}{k!\;\ell !\;(n-k-\ell )!}\cdot P^{k}\left(x\right)\;P^{\ell }\left(x^{\prime }\right) \end{array}$$

(82) $$\begin{array}{cc} \; & \;\cdot {\left[1-P\left(x\right)-P\left(x^{\prime }\right)\right]}^{n-k-\ell }\;e^{sk/n+t\ell /n}\}\\ \; & \;=\sum_{\left\{(k,\ell ):\;k+\ell \leq n\right\}}\{\frac{n!}{k!\;\ell !\;(n-k-\ell )!}\cdot {\left[P\left(x\right)\;e^{s/n}\right]}^{k}\;{\left[P\left(x^{\prime }\right)\;e^{t/n}\right]}^{\ell } \end{array}$$

(83) $$\begin{array}{cc} \; & \;\cdot {\left[1-P\left(x\right)-P\left(x^{\prime }\right)\right]}^{n-k-\ell }\} \end{array}$$

(84) $$\begin{array}{cc} \; & \;={\left[1-P\left(x\right)\;\left(1-e^{s/n}\right)-P\left(x^{\prime }\right)\;\left(1-e^{t/n}\right)\right]}^{n}. \end{array}$$

Observe that

(85) $$\begin{array}{cc} E\{\hat{P}\left(x\right)\hat{P}\left(x^{\prime }\right)\exp\left\{-u\hat{P}\left(x\right)-v\hat{P}\left(x^{\prime }\right)\right\} & =\frac{\partial^{2}\psi_{n}\left(x,x^{\prime },s,t\right)}{\partial s\;\partial t}{|}_{s=-u,\;t=-v} \end{array}$$

(86) $$\begin{array}{cc} \; & :=\psi_{n}^{\prime \prime }\left(x,x^{\prime },-u,-v\right). \end{array}$$

For $x\neq x^{\prime }$, we have

$$\begin{array}{cc} \; & E\{\hat{P}\left(x\right)\ln\hat{P}\left(x\right)\cdot \hat{P}\left(x^{\prime }\right)\ln\hat{P}\left(x^{\prime }\right)\} \end{array}$$

(87) $$\begin{array}{ll} \; & =E\left\{\hat{P}\left(x\right)\hat{P}\left(x^{\prime }\right)\int_{0}^{\infty }\int_{0}^{\infty }\frac{du\;dv}{uv}\cdot \left(e^{-u}-e^{-u\hat{P}\left(x\right)}\right)\cdot \left(e^{-v}-e^{-v\hat{P}\left(x^{\prime }\right)}\right)\right\}\\ \; & =\int_{0}^{\infty }\int_{0}^{\infty }\frac{du\;dv}{uv}\;[e^{-u-v}E\left\{\hat{P}\left(x\right)\hat{P}\left(x^{\prime }\right)\right\}-e^{-v}E\left\{\hat{P}\left(x\right)\hat{P}\left(x^{\prime }\right)e^{-u\hat{P}\left(x\right)}\right\} \end{array}$$

(88) $$\begin{array}{cc} \; & \;-e^{-u}E\left\{\hat{P}\left(x\right)\hat{P}\left(x^{\prime }\right)e^{-v\hat{P}\left(x^{\prime }\right)}\right\}+E\left\{\hat{P}\left(x\right)\hat{P}\left(x^{\prime }\right)e^{-u\hat{P}\left(x\right)-v\hat{P}\left(x^{\prime }\right)}\right\}]\\ \; & =\int_{0}^{\infty }\int_{0}^{\infty }\frac{du\;dv}{uv}\;[e^{-u-v}\psi_{n}^{\prime \prime }\left(x,x^{\prime },0,0\right)-e^{-v}\psi_{n}^{\prime \prime }\left(x,x^{\prime },-u,0\right) \end{array}$$

(89) $$\begin{array}{cc} \; & \;-e^{-u}\psi_{n}^{\prime \prime }\left(x,x^{\prime },0,-v\right)+\psi_{n}^{\prime \prime }\left(x,x^{\prime },-u,-v\right)], \end{array}$$

and for $x=x^{\prime }$,

(90) $$\begin{array}{cc} E\{{\left[\hat{P}\left(x\right)\ln\hat{P}\left(x\right)\right]}^{2}\} & =E\left\{{\hat{P}}^{2}\left(x\right)\int_{0}^{\infty }\int_{0}^{\infty }\frac{du\;dv}{uv}\cdot \left[e^{-u}-e^{-u\hat{P}\left(x\right)}\right]\cdot \left[e^{-v}-e^{-v\hat{P}\left(x\right)}\right]\right\}\\ \; & =\int_{0}^{\infty }\int_{0}^{\infty }\frac{du\;dv}{uv}\;[e^{-u-v}E\left\{{\hat{P}}^{2}\left(x\right)\right\}-e^{-v}E\left\{{\hat{P}}^{2}\left(x\right)e^{-u\hat{P}\left(x\right)}\right\} \end{array}$$

(91) $$\begin{array}{cc} \; & \;-e^{-u}E\left\{{\hat{P}}^{2}\left(x\right)e^{-v\hat{P}\left(x\right)}\right\}+E\left\{{\hat{P}}^{2}\left(x\right)e^{-\left(u+v\right)\hat{P}\left(x\right)}\right\}]\\ \; & =\int_{0}^{\infty }\int_{0}^{\infty }\frac{du\;dv}{uv}\;[e^{-u-v}\phi_{n}^{\prime \prime }\left(x,0\right)-e^{-v}\phi_{n}^{\prime \prime }\left(x,-u\right) \end{array}$$

(92) $$\begin{array}{cc} \; & \;-e^{-u}\phi_{n}^{\prime \prime }\left(x,-v\right)+\phi_{n}^{\prime \prime }\left(x,-u-v\right)]. \end{array}$$

Therefore,

(93) $$\begin{array}{cc} \mathrm{Var}\{\hat{H}\} & =\sum_{x}\int_{0}^{\infty }\int_{0}^{\infty }\frac{du\;dv}{uv}\;\left[e^{-u-v}\phi_{n}^{\prime \prime }\left(x,0\right)-e^{-v}\phi_{n}^{\prime \prime }\left(x,-u\right)-e^{-u}\phi_{n}^{\prime \prime }\left(x,-v\right)+\phi_{n}^{\prime \prime }\left(x,-u-v\right)\right]\\ \; & \;+\sum_{x^{\prime }\neq x}\int_{0}^{\infty }\int_{0}^{\infty }\frac{du\;dv}{uv}\;[e^{-u-v}\psi_{n}^{\prime \prime }\left(x,x^{\prime },0,0\right)-e^{-v}\psi_{n}^{\prime \prime }\left(x,x^{\prime },-u,0\right)\\ \; & \;-e^{-u}\psi_{n}^{\prime \prime }\left(x,x^{\prime },0,-v\right)+\psi_{n}^{\prime \prime }\left(x,x^{\prime },-u,-v\right)]-E^{2}\left\{\hat{H}\right\}. \end{array}$$

Defining (see (74) and (86))

(94) $$\begin{array}{c} Z\left(r,s,t\right):=\sum_{x}\phi_{n}^{\prime \prime }\left(x,r\right)+\sum_{x^{\prime }\neq x}\psi_{n}^{\prime \prime }\left(x,x^{\prime },s,t\right), \end{array}$$

we have

(95) $$\begin{array}{ll} \mathrm{Var}\{\hat{H}\}=\int_{0}^{\infty }\int_{0}^{\infty }\frac{du\;dv}{uv}\; & [e^{-u-v}Z\left(0,0,0\right)-e^{-v}Z\left(-u,-u,0\right)\\ & -e^{-u}Z\left(-v,0,-v\right)+Z\left(-u-v,-u,-v\right)]-E^{2}\left\{\hat{H}\right\}. \end{array}$$

To obtain numerical results, it would be convenient to particularize now the analysis to the binary symmetric source (BSS). From (75),

(96) $$\begin{array}{c} E\left\{\hat{H}\right\}=\int_{0}^{\infty }\frac{du}{u}\left[e^{-u}\;{\left(\frac{1+e^{-u}}{2}\right)}^{n-1}-e^{-un}\right]. \end{array}$$

For the variance, it follows from (84) that for $x\neq x^{\prime }$ with $x,x^{\prime }\in \left\{0,1\right\}$ and s,t∈R,

(97) $$\begin{array}{cc} \psi_{n}\left(x,x^{\prime },s,t\right)\; & ={\left(\frac{e^{s/n}+e^{t/n}}{2}\right)}^{n}, \end{array}$$

(98) $$\begin{array}{cc} \psi_{n}^{\prime \prime }\left(x,x^{\prime },s,t\right) & =\frac{\partial^{2}\psi_{n}\left(x,x^{\prime },s,t\right)}{\partial s\;\partial t}=\frac{1}{4}\left(1-\frac{1}{n}\right){\left(\frac{e^{s/n}+e^{t/n}}{2}\right)}^{n-2}e^{(s+t)/n}, \end{array}$$

and, from (87)–(89), for $x\neq x^{\prime }$

(99) $$\begin{array}{l} E\{\hat{P}\left(x\right)\ln\hat{P}\left(x\right)\cdot \hat{P}\left(x^{\prime }\right)\ln\hat{P}\left(x^{\prime }\right)\}\\ =\frac{1}{4}\left(1-\frac{1}{n}\right)\int_{0}^{\infty }\int_{0}^{\infty }\frac{du\;dv}{uv}[e^{-u-v}-e^{-\left(u/n+v\right)}{\left(\frac{1+e^{-u/n}}{2}\right)}^{n-2}\\ \;-e^{-(u+v/n)}{\left(\frac{1+e^{-v/n}}{2}\right)}^{n-2}\\ \;+e^{-(u+v)/n}{\left(\frac{e^{-u/n}+e^{-v/n}}{2}\right)}^{n-2}]. \end{array}$$

From (72), for x∈{0,1} and t∈R,

(100) $$\begin{array}{cc} \; & \phi_{n}\left(x,t\right)={\left(\frac{1+e^{t/n}}{2}\right)}^{n}, \end{array}$$

(101) $$\begin{array}{cc} \; & \phi_{n}^{\prime \prime }\left(x,t\right)=\frac{\partial^{2}\phi_{n}\left(x,t\right)}{\partial t^{2}}=\frac{e^{t/n}}{4n}{\left(\frac{1+e^{t/n}}{2}\right)}^{n-2}\left(1+ne^{t/n}\right), \end{array}$$

and, from (90)–(92), for x∈{0,1},

(102) $$\begin{array}{l} E\{{\left[\hat{P}\left(x\right)\ln\hat{P}\left(x\right)\right]}^{2}\}\\ =\frac{1}{4n}\int_{0}^{\infty }\int_{0}^{\infty }\frac{du\;dv}{uv}\;\{\left(n+1\right)e^{-u-v}-e^{-\left(u/n+v\right)}{\left(\frac{1+e^{-u/n}}{2}\right)}^{n-2}\;\left(1+ne^{-u/n}\right)\\ \;-e^{-\left(u+v/n\right)}{\left(\frac{1+e^{-v/n}}{2}\right)}^{n-2}\;\left(1+ne^{-v/n}\right)\\ \;+e^{-(u+v)/n}{\left(\frac{1+e^{-(u+v)/n}}{2}\right)}^{n-2}\;\left(1+ne^{-(u+v)/n}\right)\}. \end{array}$$

Combining Equations (93), (99), and (102), gives the following closed-form expression for the variance of the empirical entropy:

(103) $$\begin{array}{ll} \mathrm{Var}\{\hat{H}\}= & \frac{1}{2}\left(1+\frac{1}{n}\right)\int_{0}^{\infty }\int_{0}^{\infty }\frac{du\;dv}{uv}\;\left[e^{-(u+v)}-e^{-v}f_{n}\left(\frac{u}{n}\right)-e^{-u}f_{n}\left(\frac{v}{n}\right)+f_{n}\left(\frac{u+v}{n}\right)\right]\\ & +\frac{1}{2}\left(1-\frac{1}{n}\right)\int_{0}^{\infty }\int_{0}^{\infty }\frac{du\;dv}{uv}\;\left[e^{-(u+v)}-e^{-v}g_{n}\left(\frac{u}{n},0\right)-e^{-u}g_{n}\left(0,\frac{v}{n}\right)+g_{n}\left(\frac{u}{n},\frac{v}{n}\right)\right]\\ & -{\left\{\int_{0}^{\infty }\frac{du}{u}\left[e^{-u}\;{\left(\frac{1+e^{-u}}{2}\right)}^{n-1}-e^{-un}\right]\right\}}^{2}, \end{array}$$

where

(104) $$\begin{array}{cc} \; & f_{n}\left(s\right):=e^{-s}\;{\left(\frac{1+e^{-s}}{2}\right)}^{n-2}\;\frac{1+ne^{-s}}{n+1}, \end{array}$$

(105) $$\begin{array}{cc} \; & g_{n}\left(s,t\right)=e^{-s-t}\;{\left(\frac{e^{-s}+e^{-t}}{2}\right)}^{n-2}. \end{array}$$

**Figure 4:**
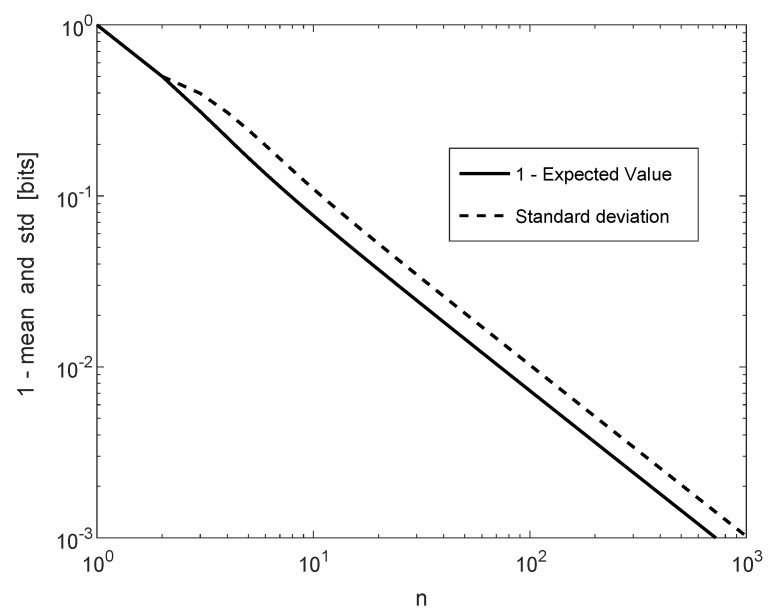
$1-E\{\hat{H}\}$ and $\mathrm{std}(\hat{H})$ for a BSS (in bits per source symbol) as a function of *n*.

For the BSS, $\ln2-E\left\{\hat{H}\right\}=E\left\{D\left(\hat{P}∥P\right)\right\}$ and the standard deviation of $\hat{H}$ both decay at the rate of $\frac{1}{n}$ as *n* grows without bound, according to Figure 4. This asymptotic behavior of $E\{D\left(\hat{P}∥P\right)\}$ is supported by the well-known result [31] (see also [18] (Section 3.C) and references therein) that for the class of discrete memoryless sources {P} with a given finite alphabet X,

(106) $$\begin{array}{cc} \; & \ln\frac{\hat{P}\left(X_{1},\ldots ,X_{n}\right)}{P(X_{1},\ldots ,X_{n})}\to \frac{1}{2}\;\chi_{d}^{2}, \end{array}$$

in law, where $\chi_{d}^{2}$ is a chi-squared random variable with *d* degrees of freedom. The left-hand side of (106) can be rewritten as

(107) $$\begin{array}{c} \ln\left(\frac{\exp\{-n\hat{H}\}}{\exp\{-n\hat{H}-nD\left(\hat{P}∥P\right)\}}\right)=nD\left(\hat{P}∥P\right), \end{array}$$

and so, $E\{D\left(\hat{P}∥P\right)\}$ decays like $\frac{d}{2n}$, which is equal to $\frac{1}{2n}$ for the BSS. In Figure 4, the base of the logarithm is 2, and therefore, $E\left\{D\left(\hat{P}∥P\right)\right\}=1-E\left\{\hat{H}\right\}$ decays like $\frac{\log_{2}e}{2n}\approx \frac{0.7213}{n}$. It can be verified numerically that $1-E\{\hat{H}\}$ (in bits) is equal to $7.25\cdot 10^{-3}$ and $7.217\cdot 10^{-4}$ for n=100 and n=1000, respectively (see Figure 4), which confirms (106) and (107). Furthermore, the exact result here for the standard deviation, which decays like $\frac{1}{n}$, scales similarly to the concentration inequality in [32] ((9)).

We conclude this subsection by exploring a quantity related to the empirical entropy, which is the expected code length associated with the universal lossless source code due to Krichevsky and Trofimov [23]. In a nutshell, this is a predictive universal code, which at each time instant *t*, sequentially assigns probabilities to the next symbol according to (a biased version of) the empirical distribution pertaining to the data seen thus far, $x_{1},\ldots ,x_{t}$. Specifically, consider the code length function (in nats),

(108) $$\begin{array}{c} L\left(x^{n}\right)=-\sum_{t=0}^{n-1}\ln Q\left(x_{t+1}|x^{t}\right), \end{array}$$

where

(109) $$\begin{array}{c} Q\left(x_{t+1}=x|x_{1},\ldots ,x_{t}\right)=\frac{N_{t}\left(x\right)+s}{t+s|X|}, \end{array}$$

$N_{t}\left(x\right)$ is the number of occurrences of the symbol x∈X in $\left(x_{1},\ldots ,x_{t}\right)$, and s>0 is a fixed bias parameter needed for the initial coding distribution (t=0).

We now calculate the redundancy of this universal code,

(110) $$\begin{array}{c} R_{n}=\frac{E\{L\left(X^{n}\right)\}}{n}-H, \end{array}$$

where *H* is the entropy of the underlying source. From Equations (108), (109), and (110), we can represent $R_{n}$ as follows,

(111) $$\begin{array}{c} R_{n}=\frac{1}{n}\sum_{t=0}^{n-1}E\left\{\ln\frac{\left(t+s|X|\right)P\left(X_{t+1}\right)}{N_{t}\left(X_{t+1}\right)+s}\right\}. \end{array}$$

The expectation on the right-hand side of (111) satisfies

(112) $$\begin{array}{l} E\left\{\ln\frac{\left(t+s|X|\right)P\left(X_{t+1}\right)}{N_{t}\left(X_{t+1}\right)+s}\right\}\\ =\sum_{x}P\left(x\right)E\left\{\ln\frac{\left(t+s|X|)P(x\right)}{N_{t}\left(x\right)+s}\right\}\\ =\int_{0}^{\infty }\left[e^{-us}\sum_{x}P\left(x\right)E\left\{e^{-uN_{t}\left(x\right)}\right\}-\sum_{x}P\left(x\right)e^{-u(s|X|+t)P(x)}\right]\;\frac{du}{u}\\ =\int_{0}^{\infty }\left[e^{-us}\sum_{x}P\left(x\right){\left[1-P\left(x\right)\left(1-e^{-u}\right)\right]}^{t}-\sum_{x}P\left(x\right)e^{-u(s|X|+t)P(x)}\right]\;\frac{du}{u}, \end{array}$$

which gives from (111) and (112) that the redundancy is given by

(113) $$\begin{array}{ll} R_{n} & =\frac{1}{n}\sum_{t=0}^{n-1}E\left\{\ln\frac{\left(t+s|X|\right)P\left(X_{t+1}\right)}{N_{t}\left(X_{t+1}\right)+s}\right\}\\ & =\frac{1}{n}\int_{0}^{\infty }\left(e^{-us}\sum_{x}P\left(x\right)\sum_{t=0}^{n-1}{\left[1-P\left(x\right)\left(1-e^{-u}\right)\right]}^{t}-\sum_{x}P\left(x\right)e^{-us|X|P(x)}\sum_{t=0}^{n-1}e^{-uP(x)t}\right)\;\frac{du}{u}\\ & =\frac{1}{n}\int_{0}^{\infty }\left[e^{-us}\sum_{x}\frac{1-{\left[1-P\left(x\right)\left(1-e^{-u}\right)\right]}^{n}}{1-e^{-u}}-\sum_{x}\frac{P\left(x\right)\;e^{-us|X|P(x)}\left(1-e^{-uP(x)n}\right)}{1-e^{-uP(x)}}\right]\;\frac{du}{u}\\ & =\frac{1}{n}\int_{0}^{\infty }\left[\frac{e^{-us}\left(\left|X\right|-\sum_{x}{\left[1-P\left(x\right)\left(1-e^{-u}\right)\right]}^{n}\right)}{1-e^{-u}}-\sum_{x}\frac{P\left(x\right)\;e^{-us|X|P(x)}\left(1-e^{-uP(x)n}\right)}{1-e^{-uP(x)}}\right]\;\frac{du}{u}. \end{array}$$

**Figure 5:**
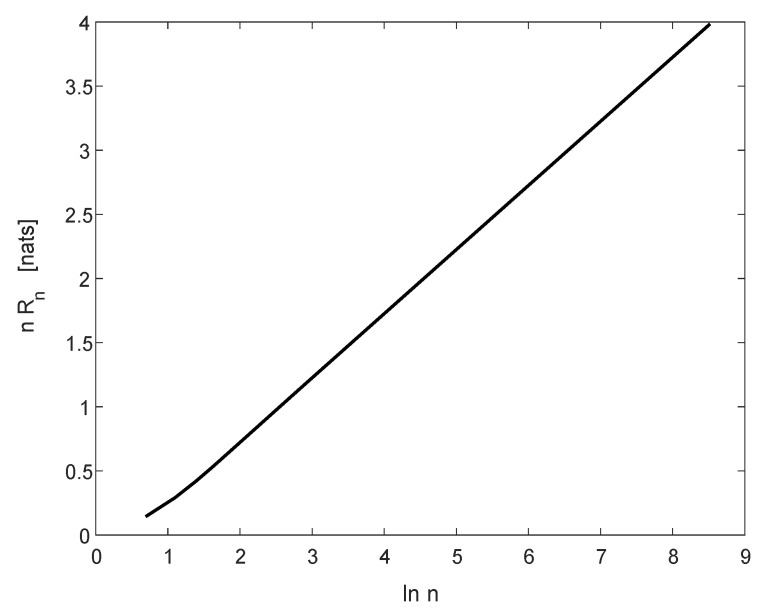
The function $nR_{n}$ vs. lnn for the BSS and $s=\frac{1}{2}$, in the range 2≤n≤5000.

Figure 5 displays $nR_{n}$ as a function of lnn for $s=\frac{1}{2}$ in the range 1≤n≤5000. As can be seen, the graph is nearly a straight line with slope $\frac{1}{2}$, which is in agreement with the theoretical result that $R_{n}∼\frac{\ln n}{2n}$ (in nats per symbol) for large *n* (see [23] (Theorem 2)).

## 4. Summary and Outlook

In this work, we have explored a well-known integral representation of the logarithmic function, and demonstrated its applications in obtaining exact formulas for quantities that involve expectations and second order moments of the logarithm of a positive random variable (or the logarithm of a sum of i.i.d. such random variables). We anticipate that this integral representation and its variants can serve as useful tools in many additional applications, representing a rigorous alternative to the replica method in some situations.

Our work in this paper focused on exact results. In future research, it would be interesting to explore whether the integral representation we have used is useful also in obtaining upper and lower bounds on expectations (and higher order moments) of expressions that involves logarithms of positive random variables. In particular, could the integrand of (1) be bounded from below and/or above in a nontrivial manner, that would lead to new interesting bounds? Moreover, it would be even more useful if the corresponding bounds on the integrand would lend themselves to closed-form expressions of the resulting definite integrals.

Another route for further research relies on [12] (p. 363, Identity (3.434.1)), which states that

(114) ∫0∞e−νu−e−μuuρ+1du=μρ−νρρ·Γ(1−ρ),Re(μ)>0,Re(ν)>0,Re(ρ)<1.

Let ν:=1, and $\mu :=\sum_{i=1}^{n}X_{i}$ where ${\left\{X_{i}\right\}}_{i=1}^{n}$ are positive i.i.d. random variables. Taking expectations of both sides of (114) and rearranging terms, gives

(115) $$\begin{array}{c} E\{(\sum_{i=1}^{n}X_{i}{)}^{\rho }\}=1+\frac{\rho }{\Gamma (1-\rho )}\int_{0}^{\infty }\frac{e^{-u}-M_{X}^{n}\left(-u\right)}{u^{\rho +1}}\;du,\;\forall \;\rho \in \left(0,1\right), \end{array}$$

where *X* is a random variable having the same density as of the $X_{i}$’s, and $M_{X}\left(u\right):=E\left\{e^{uX}\right\}$ (for u∈R) denotes the MGF of *X*. Since

(116) $$\begin{array}{c} \ln x=\lim_{\rho \to 0}\frac{x^{\rho }-1}{\rho },\;\forall \;x>0, \end{array}$$

it follows that (115) generalizes (3) for the logarithmic expectation. Identity (115), for the ρ-th moment of a sum of i.i.d. positive random variables with ρ∈(0,1), may be used in some information-theoretic contexts rather than invoking Jensen’s inequality.
